# The Story of the Insane from Year to Year

**Published:** 1902-10-11

**Authors:** 


### THE STORY OF THE INSANE FROM YEAR TO YEAR.

FIFTEENTH ANNUAL SERIES.

### CITY OF LONDON ASYLUM AT DARTFORD.

This asylum contained 512 patients at the beginning of the year, and the same number was in residence at the close of it. There were 189 first admissions, 21 readmissions, 68 discharged recovered, 59 discharged relieved, 21 discharged not improved, and 62 deaths. The average number resident was 519, and the total number under treatment was 715. Put into percentages it is seen that the recoveries were 40·48 per cent. of the admissions; and the deaths 11·94 of the average number resident, both of these percentages being above the county asylum averages. Of the year's deaths 19 were caused by cerebral and spinal diseases, 5 by consumption, 4 by pneumonia, 1 by enteritis, and 1 by enteric fever. Of the admissions 31 were supposed to owe there insanity to moral causes, intemperance in drink accounts for 31, hereditary influence for 47, and previous attacks for 49. In this asylum the number of female general paralytics is in higher proportion than is usual in county asylums, being 7 as against 9 males, and Dr. White asks whether this can be attributed to greater mental strain to which women are now subjected to by education, avocation, and the struggle for existence. It is very hard to say what may contribute to the causation of such a disease; but we doubt whether these conditions would of themselves have much influence. Considering the enormous number of suicidal cases under treatment in our county asylums, it is surprising how rarely patients succeed in accomplishing the act. At the City of London Asylum it is 14 years since a suicide occurred, but this report chronicles two instances. In the first case the patient was apparently convalescent, and when sitting at table cut his throat with a knife. The other was known to be suicidal, was under special observation, but managed to secrete a piece of a tin cigarette-box with which he killed himself. We fully agree with the Coroner's juries that none of the officials were to blame in these cases. Dr. White relates the case of a private patient who was admitted on two medical certificates and a magistrate's order; but inasmuch as the magistrate had not seen the patient she exercised her right under the Lunacy Acts of being seen by a magistrate, and this magistrate reported that he did not consider her insane. The result was that she was removed from the asylum and committed suicide within a fortnight of her discharge. Dr. White looks upon this provision of the Act as a flaw in it, and we are satisfied that he is correct in his opinion. He adds: "Thus a report by a young and inexperienced magistrate outweighs the opinions of two experts, the family medical attendant who had watched the case for months, and a fourth medical man who signed the second certificate on admission." A defect in the Act verily, and it is not the only one in that wonderful piece of legal acumen, the Lunacy Act of the 53 and 54 Vic. The asylum was visited by enteric fever and by dysenteric diarrhœa. The Visiting Committee say that the "medical superintendent continues to discharge the responsible duties of his office with ability and energy."

### LANARK DISTRICT ASYLUM AT HARTWOOD.

The number of patients in residence at the beginning of the year is not given, but there were ,807 at the end of the twelvemonths, namely, on May 31st, that being the close of their statistical year. There were 118 first admissions, 173 readmissions 87 discharged recovered, 34 discharged relieved, and 62 died. Excluding transfers, the recoveries stand at 45 per cent. of the admissions, and the deaths are 8·3 per cent. on the average number resident. Of the year's deaths 8 were caused by cerebral and spinal diseases, 10 by consumption, and 19 to other lung diseases. Of the year's admissions, 16 are stated to owe the insanity to religion, 3 to love, 30 to hereditary tendency, and 33 to intemperance in drink. During the year the late medical superintendent, Dr. Clark, died, and the committee of visitors pay a graceful tribute to his memory. "Not alone," they say, "did he possess high qualifications in that branch of his profession to which he specially devoted himself, but his powers of administration were such as to eminently fit him for the difficult duty of directing and controlling a large institution. The board recognise how much of the success of the asylum is due to Dr. Clark's unfailing energy and enthusiasm." Dr. Neil Kerr was appointed to fill the vacancy. The training of the attendants and nurses has been continued, and 12 presented themselves for the examination for the nursing certificate. Of these 11 passed, and have obtained a bonus of £2 from the committee.

